# Impact of the COVID-19 pandemic on fasting blood glucose and HbA1c levels in Lebanon: An observational analysis by age and gender

**DOI:** 10.1097/MD.0000000000044729

**Published:** 2025-10-10

**Authors:** Nadim Younes, Nada Ghorayeb, Vanda Barakett-Hamade, Marie-Hélène Gannagé-Yared

**Affiliations:** a Department of Endocrinology, Saint Joseph University, Faculty of Medicine, Beirut, Lebanon; b Laboratory Department, Hôtel Dieu de France and Faculty of Medicine, Saint Joseph University, Beirut, Lebanon.

**Keywords:** age, COVID-19, fasting blood glucose, gender, HbA1c, Lebanon

## Abstract

The impact of COVID-19 pandemic on diabetes management and control is controversial and has never been studied in Middle Eastern countries. The aim of this study is to assess recent trends in HbA1c and fasting blood glucose (FBG) in Lebanon. Data related to all the HbA1c tests performed between 2018 and 2022 were extracted from the large laboratory database of our hospital. Data related to fasting plasma glucose when measured at the same time then HbA1c were also collected. The variation in the number and values of HbA1c and FBG were analyzed according to age, gender, pandemic, sampling year and patient status (in/outpatient). The total number of HbA1c and FBG tests performed was 44,043 and 37,193 respectively. The median (interquartile) values for HbA1c and FBG were 5.55% (IQR:5.24–6.02) and 5.4 mmol/L (IQR:4.98–6.13) respectively. During the pandemic, a significant decrease in the number of HbA1c tests by 26.2% and FBG tests by 25.6% was noted, as well as a significant increase in their values (from 5.53%–5.56% and from 5.35 mmol/L to 5.43 mmol/L respectively). Median HbA1c values were higher in men than in women (5.61% vs 5.5%), in elderly subjects (5.86%) than in adults and children (5.42%, 5.16% respectively), and in inpatient than in outpatient settings (5.98% vs 5.53%), *P* < .001, for all comparisons. During the COVID-19 outbreak, the number of HbA1c and FBG tests performed decreased, but their values increased. Further studies are needed to look if this trend will last and to assess the long-term impact of these changes.

## 1. Introduction

Over the past few years, the world faced the COVID-19 pandemic which resulted in a major crisis endangering several sectors, especially the healthcare system.^[1]^ In fact, the lockdown forced people to modify their lifestyle during this period. In addition, hospital visits and in-person consultations dropped significantly, due to the fear of being exposed to the virus in a medical facility.^[2]^ Moreover, new trends in some medical practices that were not very popular before the pandemic have emerged, particularly telemedicine, which has proved to be an effective alternative.^[3]^

Worldwide, limited access to healthcare facilities and lifestyle changes during the pandemic have altered metabolic parameters in diabetic subjects.^[4]^ The rate of blood testing, whether for the diagnosis or monitoring of diabetes, has dropped significantly in several western countries such as Great Britain^[5–7]^ United States (US),^[8,9]^ Canada^[10]^ and Spain.^[11]^ This reduction was more pronounced in US,^[8,9]^ and Spain,^[11]^ probably due to a shorter time of the studies. The reduction in the number of HbA1c tests during this period in Great Britain was associated with a further deterioration in diabetes control,^[5]^ especially in subjects with elevated baseline HbA1c levels.^[6,7,12]^ A recent systemic review found evidence of a bidirectional relationship between COVD-19 and diabetes mellitus through different pathophysiological and psychosocial factors.^[12]^ Conversely, another systematic review and metanalysis showed that the pandemic worsened lipid parameters (such as triglycerides), rather than glycemic control.^[4]^ Moreover, in Lebanon, one of the worst economic crises ever documented in global history coincided with the COVID-19 outbreak.^[13]^ In the Middle East and North Africa (MENA), and particularly in Lebanon, the effects of the pandemic on HbA1c and fasting blood glucose (FBG) levels have not yet been evaluated. This evaluation is important because, in the MENA region, the prevalence of diabetes among adults aged 20 to 79 is 16.2%- one of the highest worldwide- compared to 11.3% globally.^[14]^

The aim of our study was to assess the variation in the total number of HbA1c and FBG tests performed, as well as their values over the course of the COVID-19 pandemic according to age, gender, year of sampling and patient status. In addition, the distribution of HbA1c and FBG tests in the “normal,” “prediabetes” and “diabetes” range prior to and following the COVID-19 pandemic were determined.

## 2. Materials and methods

### 2.1. Study population

Demographic and laboratory data of subjects, from all age groups, who underwent serum HbA1c with or without FBG measurement were extracted between 2018 and 2022 from the laboratory data base of Hôtel Dieu de France (HDF), one of the biggest university hospital centers in Beirut. Samples from both inpatients and outpatients were included in the analysis. The following data were collected: age, gender, sampling year and sampling setting (inpatient/outpatient). Three age groups were defined: childhood and adolescents (≤ 18 years), adults (19–64 years), and the elderly (≥ 65 years). Results were stratified according to the COVID-19 pandemic (prior to the pandemic from the beginning of 2018 to the end of 2019, following the pandemic from 2020 to the end of 2022) and according to the sampling year (beginning of 2018 to the end of 2022).

### 2.2. Hba1c measurements

Serum Hba1c quantitative determination was carried out using the turbidimetric inhibition immunoassay, and more specifically the Tina-Quant hba1c Gen. 3® test on the Roche COBAS INTEGRA platform. Patients who had a FBG test without hba1c were excluded from the study. Hba1c was categorized according to the American Diabetes Association (ADA): normal values: (<5.7%), prediabetes (values between 5.7% and 6.4%) and diabetes (values of ≥ 6.5%).^[15]^

### 2.3. FBG measurements

FBG measurements were performed using the VITROS® Chemistry analyzers (Ortho Clinical Diagnostics) which employs glucose slide technology using the glucose oxidase reaction. Blood glucose values that were not performed on a fasting state were excluded from the analysis. FBG was also categorized according to the ADA: normal values (<5.6 mmol/L), prediabetes (values between 5.6 mmol/L and 6.9 mmol/L) and diabetes (values ≥ 7 mmol/L).^[15]^

### 2.4. Ethical considerations

Informed consent to participate was judged unnecessary by our Institutional Review Board (reference of the approval CEHDF/tfem/2023/73) because the data collection was anonymous and retrospective. All methods were performed in accordance with relevant guidelines and regulations.

### 2.5. Statistical analysis

Statistical analysis was performed using SPSS software (IBM Corp; SPSS Statistics for Windows v26.1, Armonk). The distribution of quantitative variables was studied using the Kolmogorov–Smirnov and Shapiro–Wilk tests. Categorical (or qualitative) variables were expressed as percentages and frequencies, while non-normally distributed quantitative variables were expressed as median with its interquartile range (quartile 1 to 3). Spearman correlation was used to assess the association between 2 non-normally distributed quantitative variables. The χ^2^ test was used to compare proportions of categorical variables, while to compare non-normally distributed variables, the Mann-Whitney U (MWU) test was used for 2 groups, and the Kruskal–Wallis test for more than 2 groups. *P*-values <.05 were considered statistically significant.

## 3. Results

### 3.1. Population

Table 1 displays the characteristics of the study population. A total of 44,043 HbA1c tests were analyzed between 2018 and 2022. 48.3% tests were for men, and 51.7% for women. The median age of the overall population was 57 years (IQR:21–69), with a minimum of 10 months, and a maximum of 100 years. Men were older than women, without any gender significant difference (respectively 58 [IQR: 46–69] and 57 [IQR: 45–69] years, *P*-value = .055). Of the total population, 1.7% were children and adolescents, 64.4% adults and 33.9% aged 65 and over. 6.2% of tests were conducted in an inpatient setting, whereas the great majority (93.8%) were conducted as outpatients.

**Table 1:** Characteristics of the total population and by gender according to age, year of sampling, COVID-19 and patient status.

Characteristics	Total population	Men	Women
Total population	44,043	21,283 (48.3)	22,760 (51.7)
Age category			
Children–Adolescents ≤ 18 yr	741 (1.7)	356 (1.7)	385 (1.7)
Adults 19–64 yr	28,381 (64.4)	13,623 (64)	14,758 (64.8)
Elderly ≥ 65 yr	14,921 (33.9)	7304 (34.3)	7617 (33.5)
Age			
Total population (median [IQR])	57 (21–69)	58 (46–69)	57 (45–69)
0–18 yr	738 (1.7)	356 (1.7)	382 (1.7)
19–29 yr	2582 (5.8)	1139 (5.3)	1443 (6.3)
30–39 yr	4000 (9.1)	1958 (9.2)	2042 (9)
40–49 yr	6851 (15.6)	3389 (15.9)	3462 (15.2)
50–59 yr	9902 (22.5)	4699 (22.1)	5203 (22.9)
60–69 yr	9411 (21.3)	4575 (21.5)	4836 (21.2)
70–79 yr	6853 (15.6)	3376 (15.9)	3477 (15.3)
80–89 yr	3358 (7.6)	1599 (7.5)	1759 (7.7)
≥ 90 yr	348 (0.8)	192 (0.9)	156 (0.7)
Year of sampling			
2018	10,560 (24)	5120 (24.1)	5441 (23.9)
2019	9798 (22.2)	4704 (22.1)	5094 (22.4)
2020	7862 (17.8)	3888 (18.3)	3974 (17.5)
2021	7164 (16.3)	3394 (15.9)	3770 (16.5)
2022	8659 (19.7)	4177 (19.6)	4482 (19.7)
COVID-19 status			
Pre–COVID	20,358 (46.2)	9824 (46.2)	10,535 (46.3)
Post–COVID	23,685 (53.8)	11,459 (53.8)	12,226 (53.7)
Patient status			
Outpatient	41,310 (93.8)	19,622 (92.2)	21,688 (95.3)
Inpatient	2733 (6.2)	1661 (7.8)	1072 (4.7)

Data are expressed as number (percentage) or Median [IQR].

### 3.2. Number of HbA1c and FBG tests prior to and following the COVID-19 pandemic

20,358 HbA1c tests were recorded between 2018 and 2019 (10,560 in 2018 and 9798 in 2019). However, between 2020 and 2021, testing dropped to 15,026 (7862 in 2020 and 7164 in 2021), representing a decline of 26.2% (*P*-value <.001). By contrast, in 2022, the number of tests has increased to 8659 (Table 1). Similarly, for FBG, 17,148 assays were performed between 2018 and 2019 (8884 in 2018 and 8264 in 2019), while between 2020 and 2021, testing dropped to 12,750 (6732 in 2020 and 6018 in 2021), corresponding to a decline of 25.6% (*P*-value <.001). Conversely, in 2022, there was a rise in the number of tests to 7295 (Table 2).

**Table 2:** Median HbA1c and FBG values in the total population according to gender, age category, year of sampling, COVID-19, and patient status.

	Total population	Number (%)	Median (IQR)	*P*-value
		44,043	5.55 (5.24–6.02)	
HbA1c values	Sex			
	Men	21,283 (48.3)	5.61 (5.28–6.23)	<.001*
	Women	22,760 (51.7)	5.5 (5.21–5.89)	
	Age category			
	Children–adolescents	741 (1.7)	5.16 (5–5.38)	<.001†
	Adults	28,381 (64.4)	5.42 (5.16–5.77)	
	Elderly	14,921 (33.9)	5.86 (5.52–6.53)	
	Year of sampling			
	2018	10,560 (24)	5.52 (5.21–6.05)	<.001†
	2019	9798 (22.2)	5.53 (5.21–6.08)	
	2020	7862 (17.8)	5.6 (5.29–6.11)	
	2021	7164 (16.3)	5.57 (5.26–6)	
	2022	8659 (19.7)	5.52 (5.25–5.91)	
	COVID-19 status			
	Pre-COVID	20,358 (46.2)	5.53 (5.21–6.06)	<.001*
	Post-COVID	23,685 (53.8)	5.56 (5.26–6)	
	Patient status			
	Outpatient	41,310 (93.8)	5.53 (5.23–5.98)	<.001*
	Inpatient	2733 (6.2)	5.98 (5.44–7.17)	

	Total population	Number (%)	Median (Q1–Q3)	*P*-value
		37,193 (100)	5.4 (4.98–6.13)	
FBG values	Sex			
	Men	17,538 (47.2)	5.54 (5.1–6.44)	<.001*
	Women	19,655 (52.8)	5.27 (4.88–5.89)	
	Age category			
	Children–adolescents	616 (1.6)	4.91 (4.63–5.25)	<.001†
	Adults	24,141 (65)	5.26 (4.9–5.8)	
	Elderly	12,436 (33.4)	5.82 (5.24–6.93)	
	Year of sampling			
	2018	8884 (23.9)	5.3 (4.87–6.08)	<.001†
	2019	8264 (22.2)	5.4 (4.98–6.08)	
	2020	6732 (18.1)	5.4 (4.98–6.19)	
	2021	6018 (16.2)	5.43 (5.03–6.16)	
	2022	7295 (19.6)	5.45 (5.05–6.1)	
	COVID-19 status			
	Pre-COVID	17,148 (46.1)	5.35 (4.92–6.13)	<.001*
	Post-COVID	20,045 (53.9)	5.43 (5.02–6.14)	
	Patient status			
	Outpatient	35,766 (96.2)	5.38 (4.97–6.08)	<.001*
	Inpatient	1427 (3.8)	6.03 (5.13–7.9)	

FBG = fasting blood glucose; IQR = interquartile range.

* Mann–Whitney *U* test.

† Kruskal–Wallis test.

### 3.3. Analysis of HbA1c values according to gender, age, year of sampling, COVID-19 pandemic, health care services and FBG

#### 3.3.1. *HbA1c values in the overall population and according to age and gender*

**Figure 1.**
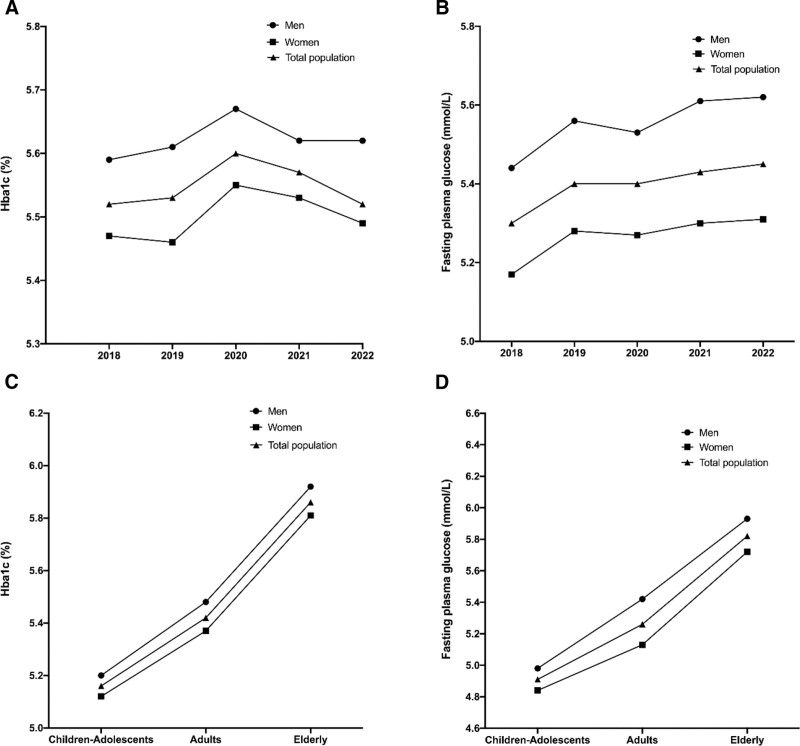
Median HbA1c and FBG in the total population, and in men and women according to years and age categories. *P* < .001 for all the comparisons between men and women and according to years and age categories. FBG = fasting blood glucose.

The median of HbA1c values in the total population was 5.55% (IQR: 5.24–6.02), with lower values in women (5.5% [IQR: 5.21–5.89]) than in men (5.61% [IQR: 5.28–6.23], *P*-value <.001, Table 2). Furthermore, a significant correlation between age and HbA1c was found (Spearman coefficient *R* = 0.489, with *P*-value <.001). Moving from 1 age group to another, HbA1c values increase: 5.16% (IQR: 5–5.38) for children and adolescents, 5.42% (IQR: 5.16–5.77) for adults, and 5.86% (IQR: 5.52–6.53) for the elderly (*P*-value <.001, Table 2). Additionally, across all age groups, men have higher HbA1c values than women with, respectively, in children–adolescents 5.2% (IQR: 5.03–5.43) vs 5.12% (IQR: 4.96–5.31) (*P*-value <. 001), in adults 5.48% (IQR: 5.2–5.95) vs 5.37% (IQR: 5.13–5.67) (*P*-value <.001), and in the elderly 5.92% (IQR: 5.53–6.65) vs 5.81% (IQR: 5.51–6.39), (*P*-value <.001 for all comparisons), Figure 1.

#### 3.3.2. *HbA1c according to year of sampling*

Over the study period, median HbA1c values varied significantly, between 5.52% and 5.6%, with the highest value in 2020 (5.6% [IQR: 5.29–6.11]) and the lowest in 2018 (5.52% [IQR: 5.21–6.05]) and 2022 (5.52% [IQR: 5.25–5.91]), *P*-value <.001, Table 2, Figure 1. This was seen in both men and women, Figure 1.

#### 3.3.3. HbA1c according to COVID-19 pandemic

A significant rise in HbA1c values was noted during the pandemic (5.56% [IQR: 5.26–6] vs 5.53% [IQR: 5.21–6.06] in pre-COVID period, *P*-value <.001, Table 2). This was noted in both men (5.6% [IQR: 5.25–6.29] pre-COVID vs 5.61% [IQR: 5.3–6.17] post-COVID) and women (5.47% [IQR: 5.18–5.89] pre-COVID vs 5.52% [IQR: 5.23–5.88] post-COVID, *P*-value <.001, Table 2).

#### 3.3.4. HbA1c values according to health care services

Most of the HbA1c measurements were performed in the outpatient setting (93.8% vs 6.2% in the inpatient setting, *P*-value <.001, Table 2). HbA1c levels measured in the inpatient setting were significantly higher than in the outpatient setting (respectively 5.98% [IQR: 5.44–7.17] vs 5.53% [IQR: 5.23–5.98], *P*-value <.001, Table 2).

#### 3.3.5. HbA1c relationship with FBG

A strong, statistically significant correlation (Spearman Coefficient *R* = 0.679, *P*-value <.001) was seen between HbA1c and FBG levels, in both men (*R* = 0.692, *P*-value <.001) and women (*R* = 0.66, *P*-value <.001).

### 3.4. Proportion of HbA1c values indicating normal, prediabetes and diabetes range

Of the total population, 26,526 (60.2%) patients had HbA1c values in the normal range, 10,483 (23.8%) in the prediabetes range, and 7034 (16%) in the diabetes range. Women had higher proportion of HbA1c in the normal range (14,711 [64.6%]) than men (11,815 [55.5%]). Conversely, the proportion of HbA1c in the prediabetes and diabetes range was higher in men (5202 [24.5%] and 4266 [20%] respectively), compared to women (5281 [23.2%] and 2768 [12.2%] respectively), (*P*-value <.001, Table 3).

**Table 3:** Prevalence of HbA1c and FBG values indicating normal, prediabetes and diabetes range according to gender, age category, year of sampling, patient and COVID-19 status.

		Normal range, number (%)	Prediabetes range, number (%)	Diabetes range, number (%)	*P*–value
HbA1c values	Total population	26,526 (60.2)	10,483 (23.8)	7034 (16)	
	Sex				
	Men	11,815 (55.5)	5202 (24.5)	4266 (20)	<.001
	Women	14,711 (64.6)	5281 (23.2)	2768 (12.2)	
	Age category				
	Children–Adolescents	652 (88)	21 (2.8)	68 (9.2)	<.001
	Adults	20,178 (71.1)	5096 (18)	3107 (10.9)	
	Elderly	5696 (38.2)	5366 (36)	3859 (25.8)	
	Year of sampling				
	2018	6454 (61.1)	2341 (22.2)	1765 (16.7)	<.001
	2019	5881 (60)	2190 (22.4)	1727 (17.6)	
	2020	4420 (56.2)	2094 (26.6)	1348 (17.2)	
	2021	4241 (59.2)	1852 (25.9)	1071 (14.9)	
	2022	5530 (63.9)	2006 (23.2)	1123 (12.9)	
	COVID-19 status				
	Pre–COVID	12,335 (60.6)	4531 (22.2)	3492 (17.2)	<.001
	Post–COVID	14,191(59.9)	5952 (25.1)	3542 (15)	
	Patient status				
	Outpatient	25,504 (61.7)	9783 (23.7)	6023 (14.6)	<.001
	Inpatient	1022 (37.4)	700 (25.6)	1011 (37)	
FBG values	Total population	22,089 (59.4)	9659 (26)	5445 (14.6%)	
	Sex				
	Men	9161 (52.2)	5149 (29.4)	3228 (18.4)	<.001
	Women	12,928 (65.8)	4510 (22.9)	2217 (11.3)	
	Age category				
	Children–adolescents	538 (87.3)	31 (5)	47 (7.7)	<.001
	Adults	16,443 (68.1)	5306 (22)	2392 (9.9)	
	Elderly	5108 (41.1)	4322 (34.7)	3006 (24.2)	
	Year of sampling				
	2018	5561 (62.6)	2006 (22.6)	1317 (14.8)	<.001
	2019	4858 (58.8)	2101 (25.4)	1305 (15.8)	
	2020	3982 (59.1)	1748 (26)	1002 (14.9)	
	2021	3514 (58.4)	1648 (27.4)	856 (14.2)	
	2022	4174 (57.2)	2156 (29.6)	965 (13.2)	
	COVID-19 status				
	Pre–COVID	10,419 (60.7)	4107 (24)	2622 (15.3)	<.001
	Post–COVID	11,670 (58.2)	5552 (27.7)	2823 (14.1)	
	Patient status				
	Outpatient	21,506 (60.1)	9320 (26.1)	4940 (13.8)	<.001
	Inpatient	583 (40.9)	339 (23.7)	505 (35.4)	

FBG = fasting blood glucose.

χ^2^ test was used for comparison.

Data are expressed as number (percentage) or Median [IQR].

The proportion of HbA1c in the normal range was higher in children and adolescents (88%), while that of prediabetes and diabetes was higher in adults (18% and 10.9% respectively) and the elderly (36% and 25.8% respectively, *P*-value <.001, Table 3).

The highest proportion of HbA1c values in the normal range was noted in 2022 (63.9%), that of prediabetes in 2020 (26.6%) and that of diabetes in 2019 (17.6%, *P*-value <.001, Table 3).

Furthermore, during the pandemic, an increase in the proportion of HbA1c values in the prediabetes range (25.1% vs 22.2% in pre-COVID) was seen, whereas before the pandemic, the proportion of HbA1c values in the normal range (60.6% vs 59.9% in pre-COVID) and diabetes range (17.2% vs 15% in pre-COVID) was greater (*P*-value <.001, Table 3).

Finally, a higher proportion of HbA1c values in the normal range was seen in the outpatient setting (61.7% vs 37.4% for inpatients), while in the inpatient setting, a higher proportion of HbA1c values in the prediabetes and diabetes range was seen (25.6% and 37% respectively, *P*-value <.001, Table 3).

### 3.5. Analysis of FBG values according to gender, age, year of sampling, COVD-19 pandemic and health care services

#### 3.5.1. FBG values in the overall population and according to gender and age

Over the study period, 37,193 FBG assays were analyzed, with the highest number in 2018 (8884, 23.9%), and the lowest in 2021 (6018, 16.2%). The median FBG value in the total population was 5.4 mmol/L (IQR: 4.98–6.13). A significant difference is observed in FBG between men and women (5.54 mmol/L [IQR: 5.1–6.44], vs 5.27 mmol/L [IQR: 4.88–5.89], *P*-value <.001, Table 2). Moving from 1 age group to another, FBG values increase: 4.91 mmol/L (IQR: 4.63–5.25) for children and adolescents, 5.26 mmol/L (IQR: 4.9–5.8) for adults, and 5.82 mmol/L (IQR: 5.24–6.93) for the elderly [(*P*-value <.001), Table 2].

#### 3.5.2. FBG according to year of sampling

From 2018 to 2022, FBG values increased, rising from 5.3 mmol/L [IQR: 4.87–6.08] in 2018 to 5.45 mmol/L [IQR: 5.05–6.1] in 2022 (*P*-value <.001, Table 2).

#### 3.5.3. FBG according to COVID-19 pandemic

The median FBG value was significantly higher after COVID-19 compared to before COVID-19 (5.43 mmol/L [IQR: 5.02–6.14] vs 5.35 mmol/L [IQR: 4.92–6.13] respectively; *P*-value <.001, Table 2).

#### 3.5.4. FBG according to health care services

Most of the FBG measurements were performed in the outpatient setting (96.2% vs 3.8% in the inpatient setting, *P*-value <.001, Table 2). The median of FBG values was higher in the inpatient setting compared to the outpatient 1 (6.03 mmol/L [IQR: 5.13–7.9] vs 5.38 mmol/L [IQR: 4.97–6.08] *P*-value <.001, Table 2).

### 3.6. Proportions of FBG values indicating normal, prediabetes and diabetes range

Of the total population, 22,089 (59.4%) patients had FBG values in the normal range, 9659 (26%) in the prediabetes range, and 5445 (14.6%) in the diabetes range.

Women had higher proportion of FBG in the normal range than men (12,928 [65.8%] vs 9161 [52.2%], *P*-value <.001). Conversely, the proportions of FBG in the prediabetes and diabetes range were higher in men (5149 [29.4%] and 3228 [18.4%] respectively), compared to women (4510 [22.9%] and 2217 [11.3%] respectively, *P*-value <.001, Table 3).

The proportion of FBG in the normal range was higher in children and adolescents (87.3%), while those of prediabetes and diabetes were higher in adults (22% and 9.9% respectively) and the elderly (34.7% and 24.2% respectively, *P*-value <.001, Table 3).

The highest proportion of FBG values in the normal range was noted in 2018 (62.6%), that of prediabetes in 2022 (29.6%) and that of diabetes in 2019 (15.8%, *P*-value <.001, Table 3).

Furthermore, during the pandemic, an increase in the proportion of FBG values in the prediabetes range (27.7% vs 24% in pre-COVID) was seen, whereas before the pandemic, the proportions of FBG values in the normal (60.7% vs 58.2% in pre-COVID) and diabetes range (15.3% vs 14.1% in pre-COVID) were greater (*P*-value <.001, Table 3).

Finally, higher proportions of FBG values in the normal and prediabetes range were observed in the outpatient setting than in the inpatient (for the prediabetes range 26.1% vs 23.7% respectively, for normal tests 60.1% vs 40.9% respectively, *P*-value <.001 (Table 3), whereas the proportion of FBG values in the diabetes range was higher in the inpatient setting (35.4% vs 13.8%, *P*-value <.001, Table 3).

### 3.7. Multilinear regression analysis with HbA1c and FPG as dependent variables

In a multilinear regression analysis, HbA1c was independently associated with sex, age, patient’s status and COVID-19 status (respectively *P* <.0001, *P* <.0001, *P* <.0001 and *P* = .027) (Table 4). Similarly, fasting plasma glucose was independently associated with sex, age, patient’s status and COVID-19 status (*P* < .0001 for all variables) (Table 4).

**Table 4:** Multiple regression analysis with HbA1c and FPG values as dependent variables.

Dependent variable HbA1c			
	Beta	Std. error	Significance
Constant	4.872	0.019	<.001
Sex	−0.212	0.01	<.001
Age	0.018	<0.001	<.001
Patient status	0.562	0.02	<.001
COVID-19 status	0.022	0.01	.027

Dependent Variable FPG			
	b	Std. error	significance
Constant	4.639	0.036	<.001
Sex	−0.416	0.019	<.001
Age	0.025	0.001	<.001
Patient status	1.195	0.048	<.001
COVID-19 status	0.067	0.019	<.001

FPG = fasting plasma glucose.

## 4. Discussion

We analyzed trends in HbA1c and FBG values over a 5-year period encompassing the COVID-19 pandemic, using data extracted from a large laboratory database at a tertiary referral hospital in Beirut.

A significant reduction in the number of HbA1c tests was observed during the pandemic period. This is most likely because, following a protracted time of lockdown, people were more concerned about doing routine checkups for their health, whereas during the pandemic, mostly ill people were being tested more frequently. Another possible explanation is that sedentary lifestyle increased during the pandemic, which may have potentially increased the HbA1c during that period.^[16]^ Worldwide, the number of primary care consultations during the pandemic period decreased drastically, and this impacted negatively the screening for early identification of chronic illnesses.^[2]^ Several studies have examined the effect of the COVID-19 pandemic on the number of HbA1c tests, but over a shorter time frame.^[8–11]^ In the US, a 66% reduction in the number of HbA1c tests was noted in the first 8 weeks of the pandemic^[9]^ and in Spain, this reduction was of 52% between March and June 2020.^[11]^ Similarly, to our results, an English study that included data gathered from 10 laboratories from December 1, 2019, to the end of 2021, revealed a decline (ranging from 7.9 to 18.1%) in the number of HbA1c tests performed in 2020 compared to those of 2019. However, in the same study, by December 2020, this number increased to 80% to 85% of the initial rate, and persisted until December 2021.^[17]^

The median HbA1c value in our population was 5.55% and significantly increased over time from 5.52% in 2018 to 5.57% in 2021. This increase was also significant when comparing the post-COVID period to the pre-COVID 1 (5.53% vs 5.56%) and occurred in both men and women.. The higher HbA1c observed during the COVID-19 pandemic could be secondary to the stress induced by the pandemic. Several studies looked at the glycemic control following the pandemic with discordant results. It has been shown that COVID-19 may have a negative impact on blood glucose levels control, switching a few normoglycemic individuals who recovered from COVID-19 infection into diabetic and a significant number of individuals into the prediabetic state.^[18]^ Also, a systematic review of 33 observational studies conducted until April 2021 on type 1 and type 2 diabetic patients showed an improvement in glycemic parameters in type 1, with a significant 0.05% fall in HbA1c. However, this was not the case in type 2 diabetes, where a significant increase in HbA1c of 0.14% was demonstrated.^[19]^ Type 2 diabetes patients might have favored unhealthy habits during their confinement. Conversely, for type 1 diabetes patients, the lockdown might have pushed them to devote more time to self-care their disease.^[19,20]^ In addition, in type 1 diabetes, parents became more involved in their children’s care as they spent more time with them at home.^[19]^ In another systematic review and metanalysis of 59 studies, no significant change in HbA1c levels was found during the post-COVID period until February 2021.^[21]^

Men had higher HbA1c than women, and the proportion of HbA1c values in the diabetes range was also significantly greater in men than in women. These data are consistent with the findings of a recent Turkish study showing this gender difference in HbA1c in adults without diabetes.^[22]^ In another study published by the WHO, and conducted in 21 Arab nations between 1990 to 2019, women had a greater prevalence of type 2 diabetes than men.^[23]^ This last finding was attributed to the higher prevalence of obesity and sedentary lifestyle among women in these countries.^[23,24]^ At the opposite, the reported prevalence of diabetes in Lebanon was of 14.5% for men and 12.2% for women in Lebanon in 2014.^[24,25]^ Finally in a recent meta-analysis investigating the relationship between gender and diabetes worldwide between 2011 and 2022, 17.7 million additional cases of diabetes in men were found.^[26]^ This gender-related difference is mainly due to larger amounts of abdominal fat in men, which results in a higher risk of developing type 2 diabetes at lower weights than in women.^[27]^

The HbA1c values increased significantly with age since our reported median value is 5.16% in children–adolescents, 5.42% in adults and 5.86% in the elderly. This finding was observed for both men and women and is consistent with other studies.^[22,23,26,27]^ Furthermore, the proportion of HbA1C value in the diabetes range was significantly higher in elderly subjects (25.8%), than in adults (10.9%) and children–adolescents (9.2%). These data are in line with the results of 2 previous studies from China^[28]^ and Turkey^[22]^ where an increase in HbA1c was observed with age^[28]^ and can be explained by an age-related decline in muscle mass, beta-cell function, glucose consumption and insulin sensitivity.^[28,29]^

Inpatients exhibited higher HbA1c values and a greater proportion of values in the diabetes and prediabetes ranges compared to outpatients. This finding is consistent with that of an Australian study, performed in 2010 which included 23,396 samples^[30]^ where the mean HbA1c was significantly higher in inpatient (5.6%) and emergency (5.5%) settings than in outpatient (5.4%) settings, even after adjustment for age. This difference is mainly explained by the fact that most outpatients are in good health. In contrast, in-hospital patients are older, with more comorbidities.^[31]^

The variation of FBG levels throughout the pandemic has not been previously investigated. Similarly, to the observed change in HbA1c values in our population, FBG significantly increased over the course of the pandemic from 5.35 mmol/L pre-COVID to 5.43 mmol/L post-COVID. In addition, FBG was significantly higher in men than in women, and in an inpatient setting compared to an outpatient 1. Moreover, the proportion of FBG values in the prediabetes and diabetes range followed the same pattern of HbA1c values throughout the pandemic. In the literature, a Malaysian study conducted on 40,667 patients between 2006 and 2012 found a highly significant correlation between HbA1c and FBG (*r*^2^ = 0.86, *P*-value <.001).^[32]^ However, a poor concordance in the diagnosis of diabetes and prediabetes between both variables was established. In fact, 30% more diagnosis of diabetes and 50% more diagnosis of prediabetes was noted with HbA1c compared to FBG.^[32]^ Additionally, another study carried out on 3 different ethnic groups of 575 patients (Chinese, Indians and Malays) showed that the relationship between FBG and HbA1c varied within populations, probably due to ethnic differences.^[33]^ Similarly, in our study, we found a strong correlation between FBG and HbA1c (Spearman Coefficient *R* = 0.679, *P*-value <.001), with a nearly equal proportions of patients in the diabetes range for both HbA1c and FBG (16% for HbA1c and 14.6% for FBG). The reason for the equal proportions observed in our population compared to other populations should be determined. The observed differences between FBG and HbA1c in diabetes diagnosis explain why the ADA recommended to combine at least 2 criteria for diagnosis and monitoring of diabetes.^[15]^

Apart from the COVID-19 pandemic and its health effects, it is important to note that since the end of 2019, Lebanon has gone through one of the worst economic crises in history.^[13]^ Lebanese people are not only facing financial problems, but also disruptions in the supply of medicines^[34]^ and the exodus of their physicians.^[35]^ This probably have led to fewer routine laboratory testing, changes in treatment and, potentially, worse diabetes control.

Our study presents certain limitations. Its cross-sectional design makes it impossible to establish causal relationships between the variables. In addition, information concerning certain clinical and biological variables that could affect HbA1c was not available, since the data were collected from the laboratory database of our hospital. These included demographic variables, such as body mass index, patient medical or surgical history (such as hematological disease, chronic renal failure, pregnancy, transfusion, splenectomy), as well as current medications. Other biological variables, such as creatinine and hemoglobin, were not included in the study. Therefore, determination of proportions of HbA1c and FBG in the normal, prediabetic or diabetic range could not be extrapolated to estimate the prevalence of prediabetes or diabetes in the Lebanese population because data about the past medical history of the individuals undergoing testing were not available...“A further limitation of our study is the inability to determine whether individuals had confirmed COVID-19 infection. Although some studies have reported modest average increases in HbA1c following SARS-CoV-2 infection,^[36]^ the long-term impact on HbA1c remains unclear.

Additionally, pandemic-related factors such as stress and dietary changes, which may influence HbA1c levels, were not assessed in our study.^[37,38]^

On the other hand, our study has several strengths. It is a very large database, covering all age groups, and in which more than 90% of the tests were performed in an outpatient setting. Analyses were carried out over a 5-year period, covering the period of the pandemic. In addition, HbA1c and FBG measurements over the study period were performed using the same assays and in the same laboratory, therefore minimizing variations secondary to assays. The HbA1c assay is NGSP-certified and standardized to the DCCT assay. This is the first study conducted in the Middle East and more particularly in Lebanon aiming to investigate the impact of the pandemic on HbA1c and FBG values. Finally, even though our study was monocentric, our hospital is a tertiary academic medical center, which serves patients from across Lebanon, making the data representative of thenational population.

## 5. Conclusion

A slight but significant increase in HbA1c and FBG after the pandemic was noted, as well as a decrease in the number of tests performed. Both HbA1c and FBG values were significantly higher in elderly subjects, men and in-hospital patients. The impact of this slight increase in HbA1c and FBG values during the pandemic in the Lebanese population should be assessed. Further studies are needed to see if this increase is long-lasting. Moreover, our findings showed that assessing the effects of gender and age is important to consider when interpreting HbA1c and FBG values, especially in the elderly population in order to reduce overdiagnosis and treatment of diabetes.

## Author contributions

**Conceptualization:** Marie-Hélène Gannagé-Yared.

**Data curation:** Nadim Younes, Marie-Hélène Gannagé-Yared.

**Formal analysis:** Nadim Younes, Marie-Hélène Gannagé-Yared.

**Methodology:** Marie-Hélène Gannagé-Yared.

**Project administration:** Marie-Hélène Gannagé-Yared.

**Resources:** Vanda Barakett-Hamade.

**Software:** Nada Ghorayeb.

**Supervision:** Marie-Hélène Gannagé-Yared.

**Validation:** Nadim Younes.

**Visualization:** Marie-Hélène Gannagé-Yared.

**Writing – original draft:** Nadim Younes.

**Writing – review & editing:** Nada Ghorayeb, Marie-Hélène Gannagé-Yared.
